# Prediction of Mefenamic Acid Crystal Shape by Random Forest Classification

**DOI:** 10.1007/s11095-022-03450-4

**Published:** 2022-12-19

**Authors:** Siya Nakapraves, Monika Warzecha, Chantal L. Mustoe, Vijay Srirambhatla, Alastair J. Florence

**Affiliations:** EPSRC CMAC Future Manufacturing Research Hub, c/o Strathclyde Institute of Pharmacy and Biomedical Sciences, Technology and Innovation Centre, 99 George Street, Glasgow, G1 1RD UK

**Keywords:** crystal shape prediction, crystallisation, mefenamic acid, random forest classification

## Abstract

**Objective:**

Particle shape can have a significant impact on the bulk properties of materials. This study describes the development and application of machine-learning models to predict the crystal shape of mefenamic acid recrystallized from organic solvents.

**Methods:**

Crystals were grown in 30 different solvents to establish a dataset comprising solvent molecular descriptors, process conditions and crystal shape. Random forest classification models were trained on this data and assessed for prediction accuracy.

**Results:**

The highest prediction accuracy of crystal shape was 93.5% assessed by fourfold cross-validation. When solvents were sequentially excluded from the training data, 32 out of 84 models predicted the shape of mefenamic acid crystals for the excluded solvent with 100% accuracy and a further 21 models had prediction accuracies from 50–100%. Reducing the feature set to only solvent physical property descriptors and supersaturations resulted in higher overall prediction accuracies than the models trained using all available or another selected subset of molecular descriptors. For the 8 solvents on which the models performed poorly (< 50% accuracy), further characterisation of crystals grown in these solvents resulted in the discovery of a new mefenamic acid solvate whereas all other crystals were the previously known form I.

**Conclusions:**

Random forest classification models using solvent physical property descriptors can reliably predict crystal morphologies for mefenamic acid crystals grown in 20 out of the 28 solvents included in this work. Poor prediction accuracies for the remaining 8 solvents indicate that further factors will be required in the feature set to provide a more generalized predictive morphology model.

**Supplementary Information:**

The online version contains supplementary material available at 10.1007/s11095-022-03450-4.

## Introduction

There is a considerable drive across the pharmaceutical industry to enhance the agility and productivity of activities involved in the development and manufacture of medicines [1]. Central interests focus on enabling faster, cost-effective drug production whilst improving sustainability and delivering improved security of supply whilst still assuring the quality and safety of medicines to patients [2, 3]. Advanced particle formation and control is an area to address as this can also enable the disruptive benefits from more closely associated knowledge across drug substance and drug product manufacturing [4]. Cyber-Physical Systems embed Industry 4.0 principles and industrial digital technologies and realise benefits from digital design [5], advanced process technology [6], and data-driven manufacturing and control such as Digital Twins [7] or medicines development and manufacture that encompass the data, models, and knowledge that describe the inter-relationships between materials, products, processes, and performance.

Crystal shape is one of the important attributes dictating the physicochemical and bulk properties of a crystalline material, which can have an impact on the process-related characteristics as well as the quality attributes of the final formulated products [8] Certain shapes of crystals are problematic during the key unit process used in the production of raw materials and downstream formulated product manufacturing. For example, needles can cause poor flowability of particulate solids and result in problems during various processes including powder flow [9], filtering [10], and tableting [11]. Therefore, the ability to routinely predict the crystal shape yielded from a given solvent could improve efficiencies in process development and medicine manufacturing and reduce the costs of research and development.

Several theoretical models are already available for crystal shape i.e. geometrical morphology based on Bravais-Friedel-Donnay-Harker (BFDH) theory [12], growth morphology based on an attachment energy calculation, the theory of Hartman-Perdok [13] or periodic bond chain (PBC) [14]. Experimental results often vary from theoretical predictions due to the influence of solvent [15, 16], impurities [17], and additives [8] in the crystallisation medium, and although progress has been made in the prediction of morphologies [18, 19], there is a need for new models that can provide practically useful, rapid prediction across a wide range of potential crystallisation environments.

In the field of crystallisation, data-driven approaches using machine learning can be powerful tools for finding relevant patterns in high-dimensional data. During the past few years, several machine learning studies showed great promise and lead to the successful discovery of novel crystal forms [20] and the successful prediction of the small molecule crystallisability [21], crystal packing [22], polymorphism, and co-crystallisation [23].

**Fig. 1:**
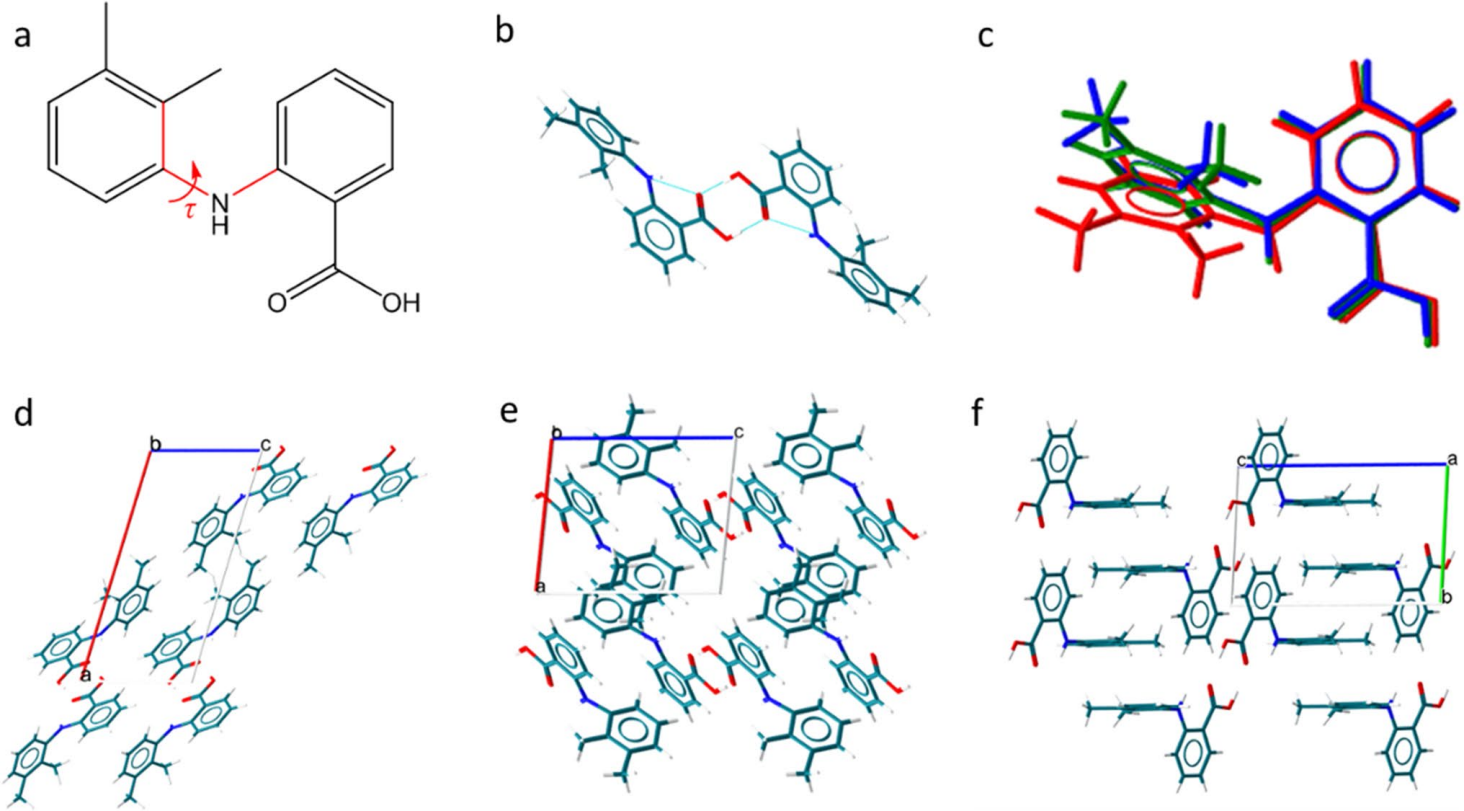
Different structures of mefenamic acid (MFA). (**a**) the molecular structure of MFA, (**b**) MFA carboxylic dimer, (**c**) the overlay of MFA molecular conformation in Form I (red, dihedral angle equal 120.0°, CCDC refcode XYANAC), Form II (blue, dihedral angle equal to 68.2°, CCDC refcode XYANAC07) and Form III (green, dihedral angle equal to 80.82°, CCDC refcode XYANAC03), the crystal structure of MFA (**d**) form I, (**e**) Form II, (**f**) Form III.

In this work, the crystal shape prediction of mefenamic acid in different solvents was investigated. Mefenamic acid (2-[(2,3-Dimethylphenyl)amino]benzoic acid, C_15_H_15_NO_2_, Fig. 1) is a high-dose analgesic drug in the non-steroidal anti-inflammatory (NSAIDs) group. It is widely used for the treatment of mild to moderate pain due to menstruation (primary dysmenorrhea) [24–26]. It is classified as a compound in class II based on the biopharmaceutical classification system (BCS) which indicates low aqueous solubility with high permeability [27, 28]. Apart from the solvated form, mefenamic acid has 3 different solid-state forms, which are forms I, II, and III [29]. During manufacturing, mefenamic acid often causes problems in processes such as granulation and tabletting because of its hydrophobicity and tendency to stick to surfaces that result from the specific crystal surface chemistry expressed. Mefenamic acid is therefore a useful example to illustrate the impact of crystal shape during drug manufacturing [30, 31] and to explore the prediction of solvent effects on crystal shape to inform subsequent process development and engineer the bulk properties of active pharmaceutical ingredients. Control of shape through appropriate particle engineering strategies can also allow the avoidance of additional downstream processing steps such as milling.

A variety of crystal shapes have been reported from prior experimental studies for mefenamic acid, ranging from plate-like to needle-like crystals [32–34]. Plates or elongated crystals of mefenamic acid were observed when crystallised from tetrahydrofuran [33], ethanol [35], ethyl acetate [30, 33], dimethylacetamide (DMA) [30, 34], and isopropanol [35], while needle-like crystals were often observed when mefenamic acid was crystallised from acetone[34, 35]. However, many studies of the crystallisation of mefenamic acid have yielded different results for crystal shape despite using the same crystallisation solvent. For example, the crystallisation of mefenamic acid from ethyl acetate carried out by Mudalip *et al*. produced needle-like crystals [34], while the SEM pictures of mefenamic acid crystallised from ethyl acetate showed plate-like crystals in the study of Panchagnula *et al*. [33] The latter study has also shown that the shape of mefenamic acid crystal grown from tetrahydrofuran and ethyl acetate changed as supersaturation levels changed [33]. Here, we’ve focused on polyhedral and needle crystal with a broad interpretation of polyhedral classification for the practical implications for downstream pharmaceutical manufacturing processes as needle-shaped crystals are more likely to cause issues during manufacturing than crystal shapes with aspect ratios closer to 1, and so are generally undesirable.

Previously, a random forest (RF) algorithm has been applied to predict the crystallisation outcomes [36, 37]. From these studies, RF performed as well as or better than other algorithms, such as support vector machines (SVM) [36, 37], deep learning multilayer perceptron networks [37], and neural networks [36].

RF has advantages over other algorithms including SVM or k-nearest neighbours which generally are more sensitive to data outliers. On the other hand, RF is robust to the outliers since its prediction relies on the averaged output from multiple independent decision trees [38]. This attribute of RF algorithm also provides a low risk of over-fitting to training data [39]. Additionally, RF also provides us with the relative ranking of variable importance which can be used to guide a feature selection and support model interpretability [40]. Therefore, in this work, we applied RF classification to predict the crystal shape of mefenamic acid as a function of recrystallisation solvent. MOE molecular descriptors were used for 30 solvents and three different sets of variables (one set that contained all available 2D descriptors, a second set that focused on molecular structure and a third set that focused on physical properties) were tested to optimise model performance. To identify which solvent descriptors were associated with RF model performance, logistic regression was applied, and variable coefficients, as well as recursive feature elimination, were considered. Powder X-ray Diffraction (PXRD) for solid-state determination and Differential Scanning Calorimetry (DSC) for thermal analysis was carried out for crystallisation from solvents which resulted in poor model performance.

## Materials and Methods

### Materials

Mefenamic acid (> 98% purity) was purchased from Merck (UK). All solvents were purchased from Fisher Scientific (UK).

### Solubility Measurements

A known amount of each type of crystalline material was added to a 1.5 ml high-performance liquid chromatography (HPLC) vial. 1 ml of a given solvent was pipetted into the pre-weighed vial containing the solid material and stirrer bar. The vial was then reweighed to determine the exact mass of solvent added and therefore the exact molar composition of the sample. Each vial was capped tightly and the cap wrapped in parafilm tape to prevent solvent loss at high temperatures. The overall weight (mg) of the sealed vial containing the solvent, stirrer, and solid material was recorded to check for weight loss after the solubility measurements in the Crystal16 Multiple Reactor (Technobis Crystallization Systems, The Netherlands). This Crystal 16 method uses the transmission of light through the vial as an indication of complete dissolution (100%) or precipitation of the crystals (less than 100%). To dissolve the particles in the stirred (700 rpm) suspension, a heating rate of 0.2 K/min was applied until a pre-set temperature was reached. For recrystallisation, the solution was cooled to a second pre-set temperature at a rate of 0.4 K/min). The temperature was kept constant for 30 min at both the pre-set low and high temperatures to ensure adequate dissolution and recrystallization. The average of the clear-point temperatures was taken as the saturation temperature for the composition in the vial. Reported solubility was calculated from the Van’t Hoff equation acquired from the Van't Hoff coordinate plot of lnC *vs* 1/T(K^−1^); where C is the concentration of mefenamic acid solution and T is the saturation temperature (see Table S1 in ESI for the solubility of mefenamic acid in all tested solvents.)

### Crystallisation

**Fig. 2:**
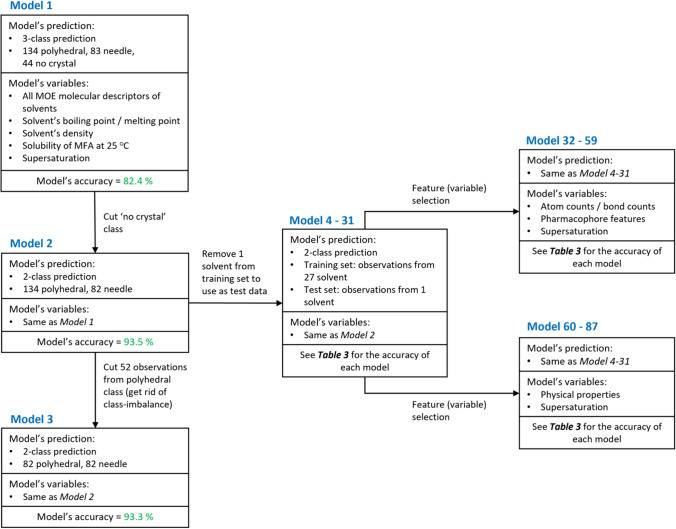
Diagram showing the dataset, variable and accuracies of all models.

Small-scale crystallisation was carried out in 20 ml scintillating vials. Appropriate amounts of mefenamic acid powder and organic solvent, as determined by the solubility experiments, were transferred into the vials. The vials were capped and covered with parafilm to avoid solvent evaporation. Vials were heated using a hot plate until all solid had visibly dissolved. To ensure no solid remained, the solution was then filtered through 0.45 µl PTFE filter discs into a clean vial. The vials were capped and placed in an incubator at 25^○^C without disturbance for 5 days. All samples were prepared in different solvents at various supersaturations for comparison and key process conditions and associated experimental outcomes recorded to provide the training set for model development and assessment (Fig. 2).

### Optical Microscopy

**Fig. 3:**
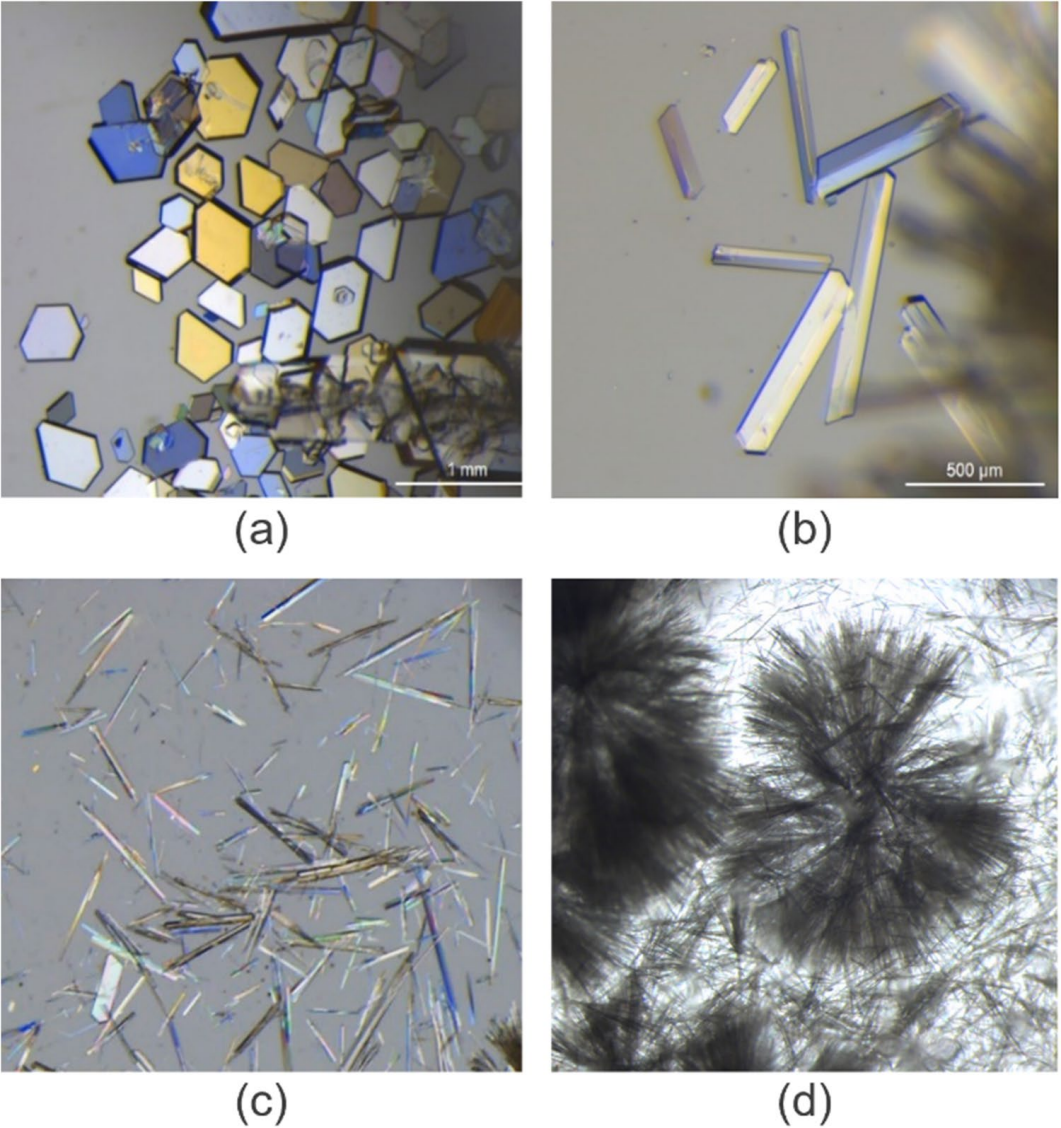
Examples of crystal shapes (**a**) plates, (**b**) elongated plates, (**c**) needles, and (**d**) spherulites. Plate and elongated plate crystals were assigned to the polyhedral class while needle and spherulitic crystals were both assigned to needle crystals.

An optical microscope (Leica M165C, supplied by Leica Microsystems (UK) Ltd.) was used for capturing two-dimensional images of the resulting crystals. The crystal shapes were manually classified into two classes: polyhedral and needle. Polyhedral crystals were comprised of any crystals with regular bounding facets including shapes such as prisms, plates and elongated crystals. Needles were defined by any sample with elongated crystals with no discernable edges or faces. Any spherulitic crystals were classed as needle crystals as they were a form of needle crystal aggregates [41]. Example images of different crystal shapes from our dataset can be seen in Fig. 3.

### Face Indexing

Single crystal X-ray diffraction (SC-XRD) was performed using D8 Venture (Bruker UK Limited), equipped with Photon III CCD detector and Cu (Copper) Kα X-ray energy source which corresponds to an x-ray wavelength of 1.5406 Å. A single crystal was prepared and fixed onto a low diffraction loop connected to a three-circle fixed Chi goniometer. The data were collected from 4° to 35° 2-theta (step size 0.017^°^) for all samples at ambient temperature. XRD on triethylamine samples was repeated in a capillary set up and the data was collected from 3° to 40° 2-theta. Face indexing was carried out using APEX3 Software to specify crystal faces.

Experimental solubility of mefenamic acid at 25°C, supersaturation levels, 2D MOE [42] solvent molecular descriptors, solvent boiling point and melting point, and solvent density were included in the dataset as input for training predictive models. Each experiment in the dataset was labelled with the crystal shape outcome. MOE descriptors used in this work were calculated from molecular structures using SMILE codes. The data was cleaned by removing the descriptors with NaN values (Not a Number, i.e. missing data) and the descriptors which contained the same value for all solvents. 206 descriptors were left in the dataset (see Table S2). From these descriptors, three different feature sets were investigated in the remaining 84 models to investigate if these different sets of solvent molecular descriptors (one set that contained all 206 available 2D descriptors, a second set of 26 descriptors that focused on molecular structure and a third set of 13 descriptors that focused on physical properties) would affect model performance. The details of the selected descriptors were listed in table S3.

### Random Forest Predictions

RF classification (Random Forest Classifier in Scikit-learn 1.0.2, Python 3.10) was applied to all models as RF have been shown to be effective for the prediction of crystallisation outcomes in previous works [21, 43, 44]. The number of decision trees was set at 100 by setting parameter n_estimators = 100 and the random state was set at 0. Other parameters were used as default values (bootstrap = True, max_depth = None, max_features = auto, max_leaf_nodes = None, min_samples_leaf = 1, min_samples_split = 2).

### Building Models

Experimental solubility of mefenamic acid at 25°C, supersaturation levels, 2D MOE solvent molecular descriptors, solvent boiling point and melting point, and solvent density were included in the dataset as input for training predictive models. Each experiment in the dataset was labelled with the crystal shape outcome. MOE descriptors used in this work were calculated from molecular structures using SMILE codes. After data cleaning by removing the descriptors with NaN value (missing data) and the descriptors which contain the same value for all solvents, 206 descriptors were left in the dataset (see Table S2 for details of descriptors). From this dataset, 87 models were built to assess the optimum performance for predicting crystal shape. The different considerations and test criteria used for these models are shown in Fig. 2.

Model 1 used the entire dataset for 3-class prediction as follows: polyhedral (134 observations), needle (83 observations), and no crystal (44 observations). The class of no crystal was then removed from the datasets for all remaining models due to the relatively low occurrence of this outcome. As class imbalance was present in the dataset used for Model 2, some observations in the polyhedral class were removed from the dataset used in Model 3 to remove class imbalance. To remove class imbalance, observations were removed to maintain the spread of original data (i.e. data points for solvents with low numbers of observations were kept in the dataset while some data points for the solvents with higher numbers of observations were removed) rather than random selection.

From 206 descriptors, feature selection was applied to the final 84 models to investigate if different sets of solvent molecular descriptors (one set that contained all available 2D descriptors, a second set that focused on molecular structures and a third set that focused on physical properties) would affect model performance. Atom counts & bond counts and pharmacophore feature MOE subsets were grouped as these descriptors directly reflect the structure and connectivity of the solvent molecules. By comparison, the physical properties MOE subset comprises of descriptors that describe the way the solvent interacts with surrounding molecules (such as molecular weight, polarizability, refractivity, mass density, and aqueous solubility). Further information on the MOE descriptor subsets available can be found in the ESI. The details of the selected descriptors were listed in Table S3.

### Model Evaluation

Train-test split and n-fold cross-validation [45] were used to evaluate the prediction accuracy of the RF classification models. Table I shows the prediction accuracy of the models evaluated with different ratios of training and test data. Ratios of 75:25, 80:20, and 90:10 were used in the train-test split method, comparable to fourfold, fivefold, and tenfold cross-validation, respectively.

**Table I:** Model evaluation by train-test split and cross-validation of models 1, 2 and 3. SD = Standard Deviation

Prediction	Accuracy by train-test split (train:test)			Accuracy by cross-validation		
	75:25	80:20	90:10	fourfold	fivefold	tenfold
Model 1 (3 classes)	84.4% (SD = 3.6%)	84.2% (SD = 4.5%)	85.0% (SD = 6.2%)	82.4% (SD = 3.1%)	84.7% (SD = 2.1%)	83.1% (SD = 4.6%)
Model 2 (2 classes w/ class-imbalance)	91.8% (SD = 3.3%)	92.1% (SD = 3.6%)	93.7% (SD = 4.6%)	93.5% (SD = 2.1%)	94.4% (SD = 2.4%)	93.5% (SD = 4.7%)
Model 3 (2 classes w/o class-imbalance)	93.8% (SD = 3.8%)	93.6% (SD = 4.3%)	95.5% (SD = 4.7%)	93.3% (SD = 5.3%)	92.6% (SD = 6.4%)	95.7% (SD = 4.7%)

Overall, the different accuracies as calculated by either train-test split or cross-validation varied by no more than 3%. This consistency shows the RF approach to be robust to different methods of validation. The lowest ratio was used to save computational time and reduce standard deviation in the model [45]. Between the two evaluation methods, the variance of the accuracy calculated from n-fold cross-validation was lower than those from the train-test split. As a result, fourfold cross-validation was used for evaluating the model performance in this work.

## Results and Discussion

### Crystallisation

MFA was crystallised from 30 solvents over 5 days at a range of supersaturations (261 observations in total). Crystallisation was observed in all solvents except isobutyl acetate and 1-butanol during the 5-day experimental period. Table II presents crystal shapes and corresponding solvents. Four crystal morphologies were observed: plates, elongated plates, needles, and spherulites (Fig. 3). Plates (Fig. 3a) and elongated plates (Fig. 3b) were considered as polyhedral crystals while needle (Fig. 3c) and spherulitic (Fig. 3d) crystals were both considered as needle crystals. Based on face-indexing data, the biggest face which dominated the polyhedral crystal is [100] (Figure S1). This observed crystal shape corresponded to the BFDH morphology of mefenamic acid crystal form-I (Figure S2).

**Table II:** The list of organic solvents categorized by the shape of mefenamic acid crystals they can produce

Polyhedral	Needle	Supersaturation dependent (polyhedral supersaturation range, needle supersaturation range)
1,2 dichloroethane	1-bromobutane	1,4 dioxane (1.18 – 1.28, 1.39 – 1.91)
1-chlorobutane	1-methylnaphtalene	2-butanol (1.51 – 1.83, 1.94 – 2.03)
1-octanol	aniline	2-butanone (1.10 – 1.50, 1.60 – 2.01)
2-methoxyethanol	anisole	2-propanol (1.14 – 1.41, 1.49 – 1.99)
acetic acid	methyl acetate	butyl acetate (1.32, 1.42 – 2.00)
acetone	nitromethane	diethyl sulfide (1.06 – 1.57, 1.76 – 1.94)
acetonitrile	toluene	Methanol (1.13 – 1.22, 1.30 – 1.98)
chloroform		
ethanol		
DMF		
ethyl acetate		
iodomethane		
triethylamine		
trichloroethylene		

Polyhedral crystals were always found at all supersaturation levels (in the range of 1.1 – 2.7) when using the following solvents: 1,2 dichloroethane, 1-chlorobutane, 1-octanol, 2-methoxyethanol, acetic acid, acetone, acetonitrile, chloroform, ethanol, DMF, ethyl acetate, iodomethane, triethylamine, trichloroethylene. At a supersaturation range of 1.1 – 3.0, the crystals of mefenamic acid exhibited needle shape when crystallised from the following solvents: 1-bromobutane, 1-methylnaphtalene, aniline, anisole, methyl acetate, nitromethane, toluene. As for crystals grown from 1,4 dioxane, 2-butanol, 2-butanone, 2-propanol, butyl acetate, diethyl sulphide and methanol, the crystal shape was supersaturation dependent. For these solvents, polyhedral crystals were observed at low supersaturation and needles were observed at higher supersaturations.

At low supersaturation levels, mefenamic acid did not crystallised in some tested solvents within 5 days. These samples were labelled as ‘no crystal’. See the detail of the samples in ‘no crystal’ class in ESI, Table S2).

### Model Performance Using Crystal Shape Observations from all Solvents in the Training Set

Three RF classification models were built initially to determine the efficacy of this method and understand the extent to which the class imbalance present in the dataset would affect prediction accuracies. In Model 1 the full dataset was separated into the following 3 classes: polyhedral (134 data points), needle (83 data points), and ‘no crystal’ (44 data points). In Model 2, the ‘no crystal’ class was removed resulting in a 2-class prediction model. The class-imbalance present in Model 2 was removed for the dataset used in Model 3 by removing observations in the polyhedral class until the needle and polyhedral classes were equally populated. For fourfold cross-validation, Model 1 had the lowest performance accuracy (82.4%) while Models 2 and 3 had performance accuracies of 93.5% and 93.3%, respectively. Additionally, the values of accuracy, precision, recall, and F1-score of these three models also agreed with the model accuracies (Table III). As these results indicate that the class imbalance observed in Model 2 did not noticeably affect the model performance, the dataset used in Model 2 was used all for further models with the modifications discussed below.

**Table III:** The models’ precision, recall, and F1-score. The ‘support’ column indicates the number of test data in each crystal class

Model prediction	Precision	Recall	F1-score	Support
Model 1 (3 crystal outcomes with class imbalance)				
Polyhedral	0.83	0.94	0.88	31
Needle	0.89	0.80	0.84	20
No crystal	0.85	0.73	0.79	15
Model 2 (2 crystal outcomes with class imbalance)				
Polyhedral	0.91	1.00	0.95	31
Needle	1.00	0.87	0.93	23
Model 3 (2 crystal outcomes without class imbalance)				
Polyhedral	1.00	0.84	0.91	19
Needle	0.88	1.00	0.94	22

### Prediction of Crystal Shape From Solvents not Included in the Training Set

To determine the ability of this methodology to predict crystal morphology from solvents for which no data was present in the training set, we built 84 additional models that each had all observations for a single solvent removed from the training data. The performance accuracy for each model was then assessed using the crystal morphologies for the solvent excluded from the training data. Additionally, three different feature sets were tested to determine if model performance accuracy was affected by the inclusion of different variables in the training sets (see Fig. 2 and Table IV for more details). The three feature sets were (i) solvent physical properties and supersaturations of crystallisation experiments (ii) atom count, bond count, pharmacophore descriptors for the solvents and supersaturations of the crystallisation experiments and (iii) all features present in the first two feature sets.

**Table IV:** The prediction accuracy of the models testing the prediction of crystal shape from individual solvents

Solvent in which test set data was collected	Number of samples in test set	Experimental crystal shape	Solvent descriptors					
			Variable group 1: All solvent descriptors		Variable group 2: Atom counts / bond counts + pharmacophore features		Variable group 3 Physical properties	
			Predicted shape	Prediction accuracy	Predicted shape	Prediction accuracy	Predicted shape	Prediction accuracy
1,2-dichloroethane	7	Polyhedral	Polyhedral	100%	Polyhedral	100%	Polyhedral	100%
Chloroform	5	Polyhedral	Polyhedral	100%	Polyhedral	100%	Polyhedral	100%
Trichloroethylene	4	Polyhedral	Polyhedral	100%	Polyhedral	100%	Polyhedral	100%
Ethanol	9	Polyhedral	Polyhedral	100%	Polyhedral	100%	Polyhedral	100%
Aniline	7	Needle	Needle	100%	Needle	100%	Needle	100%
Anisole	10	Needle	Needle	100%	Needle	100%	Needle	100%
Toluene	6	Needle	Needle	100%	Needle	100%	Needle	100%
Acetonitrile	12	Polyhedral	Polyhedral	100%	Polyhedral	100%	10 poly, 2 nd	83.3%
Acetone	9	Polyhedral	7 poly, 2 nd	77.8%	Polyhedral	100%	Polyhedral	100%
Iodomethane	3	Polyhedral	Polyhedral	100%	1 poly, 2 nd	33.3%	Polyhedral	100%
2-propanol	10	6 poly, 4 nd	polyhedral	60.0%	polyhedral	60.0%	7 poly, 3 nd	90.0%
2-methoxyethanol	10	Polyhedral	4 poly, 6 nd	40.0%	6 poly, 4 nd	60.0%	Polyhedral	100%
2-butanol	6	3 poly, 3 nd	1 poly, 5 nd	66.7%	1 poly, 5 nd	66.7%	1 poly, 5 nd	66.7%
2-butanone	9	5 poly, 4 nd	polyhedral	55.6%	polyhedral	55.6%	6 poly, 3 nd	88.9%
1-methylnaphthalene	8	Needle	needle	100%	needle	100%	polyhedral	0%
Methanol	10	6 poly, 4 nd	polyhedral	60.0%	polyhedral	60.0%	polyhedral	60.0%
Diethyl sulfide	7	5 poly, 2 nd	polyhedral	71.4%	polyhedral	71.4%	needle	28.6%
1,4-dioxane	8	2 poly, 6 nd	6 poly, 2 nd	50.0%	needle	75.0%	polyhedral	25.0%
DMF	9	Polyhedral	3 poly, 5 nd	33.3%	needle	0%	polyhedral	100%
Ethyl acetate	6	Polyhedral	needle	0%	3 poly, 3 nd	50.0%	4 poly, 2 nd	66.7%
Acetic acid	10	Polyhedral	1 poly, 9 nd	10.0%	needle	0%	polyhedral	100%
Butyl acetate	7	1 poly, 6 nd	polyhedral	14.3%	polyhedral	14.3%	3 poly, 4 nd	71.4%
1-bromobutane	7	Needle	polyhedral	0%	polyhedral	0%	2 poly, 5 nd	71.4%
1-chlorobutane	6	Polyhedral	needle	0%	1 poly, 5 nd	16.7%	2 poly, 4 nd	33.3%
Triethylamine	8	Polyhedral	2 poly, 6 nd	25.0%	2 poly, 6 nd	25.0%	needle	0%
1-Octanol	7	Polyhedral	needle	0%	needle	0%	needle	0%
Methyl acetate	11	Needle	polyhedral	0%	polyhedral	0%	polyhedral	0%
Nitromethane	5	Needle	polyhedral	0%	polyhedral	0%	polyhedral	0%

Poly, polyhedral crystals; nd, needle. All training set and test set data included the relevant solvent descriptors and experimental supersaturation as x values and crystal shape labels as y values

In total, 32 out of 84 models predicted the shape of mefenamic acid crystals with 100% accuracy, and the models trained with the first feature set resulted in the best overall prediction accuracy for morphologies across all solvents. The results explained here are tabulated in Table IV for clarity. When including only physical property descriptors and supersaturations in the model features, 12 solvent models had 100% prediction accuracy, 8 solvent models had accuracies from 50–100%, and the remaining 8 models had prediction accuracies below 50%. When using atom count, bond count, and pharmacophore descriptors as variables, 10 models had 100% prediction accuracy, 7 models had accuracies from 50–100%, and 11 models had accuracies below 50%. For the models using all solvent molecular descriptors as variables, 10 models had 100% prediction accuracy, 6 models had accuracies from 50–100%, and 12 models had accuracies below 50%. Thus, using all descriptors in the feature set resulted in the lowest performance across all solvents while using only solvent physical properties and supersaturations as the feature set had the highest accuracies across all solvents. These results suggest that some of the variables in the atom count, bound count and pharmacophore descriptor feature set had a confounding effect on model performance.

Accuracy trends were also observed for solvent type. All models had high prediction accuracies for morphologies of crystals grown in chlorinated solvents (1,2 chloroethane, chloroform, and trichloroethylene), aniline, anisole, ethanol, and toluene. By contrast, the models performed poorly when predicting morphologies for crystals grown from 1-octanol, triethylamine, methyl acetate, and nitromethane. Model performance was determined by using an external test set comprised of multiple experiments conducted at various supersaturations in the solvent not included in the training data. To understand why RF classification consistently performed well for some solvents and badly for others, these results were explored via logistic regression. Crystal form characterisation was also investigated for crystals grown in solvents where morphology was poorly predicted.

### Variable Importance in the RF Classification for Crystal Morphology Prediction

Table V shows the two most important variables for each model for solvents with the highest and lowest prediction accuracies. For the first two variable sets, the most important feature focus on the structure of the molecule, mainly the number of rings, number of rigid or single bonds, atom count and adjacency matrix. While there is no clear difference between the most important descriptors identified for the models that performed poorly or well using Variable Groups 1 & 2, we do observe some difference in the top two important variables for models trained with Variable Group 3. The models trained using these two sets of variables performed similarly in terms of the number of correct and incorrect predictions. Models using the third variable set (13 physical properties MOE descriptors) performed much better and identified the most important variables including aqueous solubility and molecular refractivity. Unlike the models that performed well, 3 of the 5 models that performed poorly using the third variable set identified features related to van der Waals volume as the most important. As relative feature importance alone is insufficient to describe the variability in model performance for different solvents, this will be explored further by logistic regression later in the paper.

**Table V:** List of first and second most important variables of the models for predicting the shape of crystals crystallised from individual solvents

Crystallisation solvents	The most important variables of each model		
	Variable group 1: All solvent descriptors	Variable group 2: Atom counts / bond counts + pharmacophore features	Variable group 3: Physical properties
Solvents where the crystals were 100% accurately predicted by the models			
1,2-Dichloroethane	1. number of rings 2. adjacency matrix	1. no. of rigid bonds 2. atom count	1. aqueous solubility 2. molecular refractivity
Chloroform	1. adjacency matrix 2. number of rings	1. no. of rigid bonds 2. no. of single bonds	1. aqueous solubility 2. molecular refractivity
Ethanol	1. adjacency matrix 2. number of rings	1. no. of rigid bonds 2. no. of single bonds	1. aqueous solubility 2. molecular refractivity
Trichloroethylene	1. adjacency matrix 2. number of rings	1. no. of rigid bonds 2. no. of single bonds	1. aqueous solubility 2. molecular refractivity
Aniline	1. adjacency matrix 2. number of rings	1. no. of single bonds 2. no. of rigid bonds	1. aqueous solubility 2. bpol^#^
Anisole	1. number of rings 2. distance Matrix	1. no. of rigid bonds 2. no. of single bonds	1. aqueous solubility 2. molecular refractivity
Toluene	1. adjacency matrix 2. number of rings	1. no. of single bonds 2. no. of rigid bonds	1. aqueous solubility 2. molecular refractivity
Solvents where the crystals were incorrectly predicted by the models			
1-Chlorobutane	1. number of rings 2. adjacency matrix	1. no. of rigid bonds 2. no. of single bonds	1. aqueous solubility 2. bpol^#^
1-Octanol	1. chi1_C* 2. zagreb^$^	1. no. of heavy atoms 2. no. of rigid bonds	1. aqueous solubility 2. van der Waals volume
Triethylamine	1. distance matrix 2. molecular refractivity	1. no. of single bonds 2. no. of rigid bonds	1. molecular refractivity 2. van der Waals volume
Methyl acetate	1. distance matrix 2. adjacency matrix	1. no. of rigid bonds 2. no. of rings	1. van der Waals volume 2. molecular refractivity
Nitromethane	1. adjacency matrix 2. number of rings	1. no. of rigid bonds 2. atom count	1. bpol^#^ 2. aqueous solubility

We also observed that if the model did not identify one of the two most important variables as aqueous solubility or molecular refractivity, the accuracy of the predictions was low. Aqueous solubility can be linked with the ability of the molecules to form H-bonds while molecular refractivity is related to London dispersive forces [46]. The anisotropy of the rate of incorporation of growth units from solution to individual crystal faces determines crystal shape [8, 47]. In solution, both the crystal surface and solute growth units are solvated, and the relative growth rates of faces depend on the strengths of intermolecular interactions between the solute–solvent and solvent-crystal surfaces [48, 49]. It was demonstrated previously that the crystallisation from organic solvents is dominated by weak interactions between permanent dipoles and London dispersion forces between the nonpolar groups of the solute and solvent and these interactions are responsible for different crystal shapes obtained from various solvents [50]. Our machine-learning model also identified these interactions as the most important distinguishers between models for solvents that show very good prediction accuracy (100%). Further exploration of feature importance can be found in the ESI where model performance was investigated for variations on Model 2 (no solvent removal) trained on supersaturation and only one additional feature. This analysis also indicated that molar refractivity and aqueous solubility are key features in these models.

### Using Logistic Regression to Understand Model Performance

As seen in Table V, we see that changes in the relative feature importance were not sufficient to explain the variable model performance for the different solvents. Thus, logistic regression was also used to probe why the RF models consistently performed well for some solvents and poorly for others even when the solvent feature sets were changed (Table VI). For this analysis, models 60–87 were used (i.e. solvent-exclusion models that used solvent physical properties and supersaturation as training variables), and models with prediction accuracy greater than 50% were labelled as 1 while models with prediction accuracies less than 50% were labelled as 0. This set of models was chosen as the feature set for these models resulted in the highest overall prediction accuracy across solvents. The most important features in logistic regression can be determined by the highest absolute values of the variable coefficients and/or recursive feature elimination until only the most relevant features remain.

**Table VI:** MOE descriptors included as variables in the RF classification models 60–87 listed according to importance scores in the logistic regression analysis of the performance of these models. RF model accuracies above 50% were labelled as 1 in the logistic regression analysis while RF model accuracies below 50% were labelled as 0. Recursive feature elimination was done until the 6 most relevant features/variables remained (these 6 features are ranked as 1 in the Table below)

MOE descriptor	Summary of MOE descriptor	Logistic regression coefficients	Ranking by recursive feature elimination
bpol	sum of the absolute value of the difference between atomic polarizabilities of all bonded atoms in the molecule	-0.7288	1
apol	sum of the atomic polarizabilities	-0.4332	1
logS	log of the aqueous solubility (mol/L)	0.3232	1
SMR	molecular refractivity	-0.2926	1
vdw_area	Area of van der Waals surface	-0.2872	1
vdw_volume	van der Waals volume	-0.2594	1
mr	molecular refractivity	-0.2587	2
logP(o/w)	log of the octanol/water partition coefficient	-0.2248	3
density	molecular mass density	0.1845	4
reactive	indicator of the presence of reactive groups	0.1039	5
TPSA	polar surface area	-0.1081	6
SlogP	log of the octanol/water partition coefficient	-0.0897	7
Weight	molecular weight	-0.0209	8

From the relative importance of different variables in the logistic regression analysis, we see that polarizability (apol, bpol) and solubility (logS) play an important role in determining whether the RF classification model performed well for a given solvent. While the polar surface area variable (TPSA) was deemed a relatively unimportant feature, this rating may be due to this variable being redundant after the inclusion of apol and bpol into the models. Variables pertaining to van der Waals interactions (vdw_area and vdw_volume) were also amongst the more relevant features in determining whether the RF classification models performed well for observations in a given solvent. As we would expect crystal morphologies to be strongly influenced by intermolecular interactions between the mefenamic acid and the crystallisation solvent, the importance of variables pertaining to solubility, polarity and van der Waals interactions corresponds with the important physical parameters in a crystallisation experiment. According to these results, the values of features related to polarizability and aqueous solubility can dictate whether or not a model performs well. Thus, while this methodology may work well for solvents with a given polarizability or aqueous solubility calculated descriptors, additional features may be needed to improve the model performance for solvents with higher/lower values of solubility or polarizability. Further work could explore what feature values are associated with better performance and what additional information could be included to improve the model performance for these models. This further work would also benefit from a larger dataset of solutes and solvents on which to test these hypotheses.

### Characterisation of Mefenamic Acid Crystals Grown in Triethylamine

**Fig. 4:**
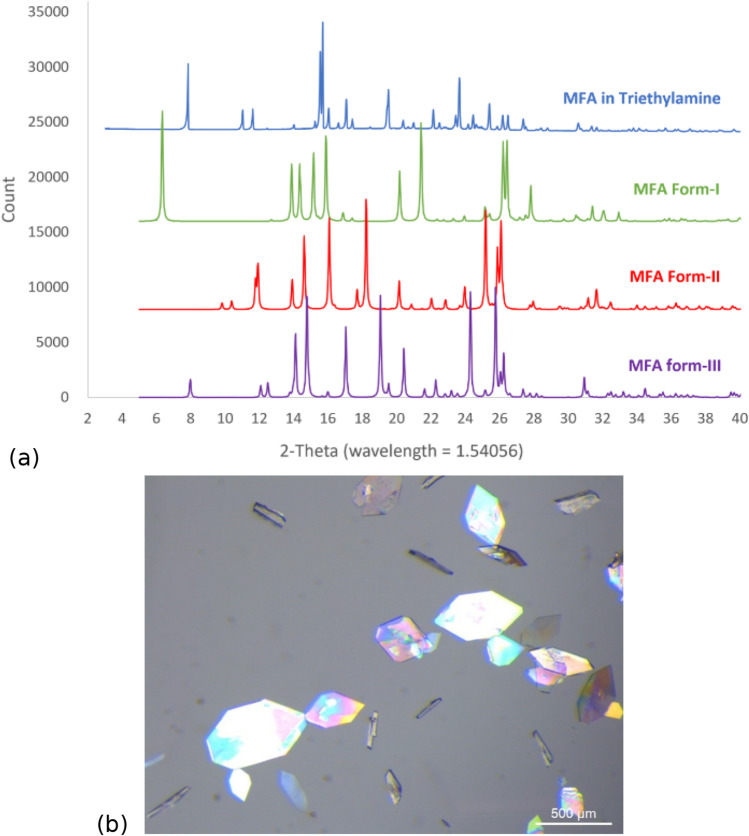
(**a**) Experimental powder X-ray diffraction pattern of mefenamic acid crystallised from triethylamine, compared to the simulated powder patterns of mefenamic acid form-I (refcode: XYANAC), II (refcode: XYANAC02), and III (refcode: XYANAC03) calculated from Mercury, (**b**) Mefenamic acid crystals crystallised from triethylamine at supersaturation = 1.4.

Further crystal characterisation was done for the crystals grown in solvents with the models showing low prediction accuracy. All samples were consistent with mefenamic acid form I (See PXRD patterns in ESI, Figure S4) except the sample crystallised from trimethylamine which exhibited a notably distinct PXRD pattern (Fig. 4a). Characterisation of the mefenamic acid grown in triethylamine was of particular interest as results revealed these crystals to be a previously unidentified solvate of mefenamic acid. Additionally, the shape of the crystals grown in triethylamine had thinner flat plates as observed under a microscope when compared to the plate crystals of mefenamic acid form-I crystallised from the other solvents (see Fig. 4b).

Characterisation of these crystals by differential scanning calorimetry (DSC) also suggested that mefenamic acid crystals grown from triethylamine were a previously unidentified solvate (see ESI for details).

## Conclusions

The choice of solvent in crystallisation is a critical design decision and can affect the crystal morphology with further implications for downstream manufacturability. For this work, we generated 261 experimental observations of MFA crystal shape in 30 various organic solvents at the range of supersaturation levels between S = 1.0 – 3.0. RF classification models can predict the shape of mefenamic acid crystals observed from different solvents experimentally. Thus, the results illustrate that RF classification can be a useful tool to predict the experimental crystal shape of MFA. Our two-class RF prediction model with polyhedral and needle classes resulted in a prediction accuracy of 93%. This model was further modified (as detailed in Fig. 2) to explore prediction accuracies for crystals grown in specific solvents. For solvents that were excluded from the training set at all supersaturation levels, the prediction accuracy depended on the solvent. The most important variables for the correctly predicted solvents relate to H-bonds and London dispersion forces identifying this interaction as key for the determination of a crystal shape. Additionally, to improve the capability of the predictive models, further model development could include exploring different sets of molecular descriptors, optimising hyperparameters and investigating more compounds, solvents, and crystallisation parameters.

Whilst demonstrated only for mefenamic acid it is expected that with the appropriate data, the application of this tool can be broadened to cover a wider range of active ingredient molecular and crystal attributes. Such data are already often collected during physical form selection, solubility and early development studies. Hence, this study highlights the potential role of machine learning and data-driven predictive tools to support decision making during pharmaceutical process development. Informing solvent selection, reducing experimental time and material consumption and enabling the selection of conditions that deliver materials engineered to achieve desirable attributes.

## Supplementary Information

Below is the link to the electronic supplementary material.

Supplementary file1 (DOCX 1376 KB)

## Data Availability

The datasets generated during and/or analysed during the current study are available in the University PURE system. The code for this work can be found at https://github.com/SiyaNakapraves/Random-forest-classification-for-the-prediction-of-mefenamic-acid-crystal-shapes.
