# Supplementary material for: Prediction of Mefenamic Acid Crystal Shape by Random Forest Classification
Source: Pharm Res. 2022 Dec 19;39(12):3099–111. doi: 10.1007/s11095-022-03450-4 (PMC9780130; doi:10.1007/s11095-022-03450-4)
Supplement: Supplementary file 1 — Supplementary file1 (DOCX 1376 KB) [file 11095_2022_3450_MOESM1_ESM.docx]

**Supplementary Information for Prediction of crystal shape of mefenamic acid using machine learning**

Siya Nakapraves,^1^ Monika Warzecha,^1^ Chantal L. Mustoe,^1^ Vijay Srirambhatla,^1^ and Alastair J. Florence,^1,*^

*^1^EPSRC CMAC Future Manufacturing Research Hub, c/o Strathclyde Institute of Pharmacy and Biomedical Sciences, Technology and Innovation Centre, 99 George Street, Glasgow, G1 1RD, U.K.*

[**alastair.florence@strath.ac.uk*](mailto:*alastair.florence@strath.ac.uk)

***The solubility of mefenamic acid in the tested organic solvents***

The solubility of mefenamic acid was tested in the following organic solvents presented in **Table S1**; using Crystal16 Multiple Reactor (Technobis Crystallization Systems).

**Table S1** The solubility of mefenamic acid in organic solvents calculated from Van’t Hoff equation acquired from the Van't Hoff coordinate plot of lnC vs 1/T(K ^-1^)

| Solvent | Van’t Hoff equation | Solubility (25 °C) [mg/mL] |
| --- | --- | --- |
| 1-Octanol | y = -3,403.7746x + 13.3327 | 6.76 |
| Tetrahydrofuran | y = -1,568.6529x + 10.3958 | 169.33 |
| Trichloroethylene | y = -4,568.4531x + 16.2482 | 2.50 |
| Nitromethane | y = -2,854.7814x + 9.8773 | 1.35 |
| Iodomethane | y = -5,129.5747x + 17.8226 | 1.84 |
| Butyl acetate | y = -2,823.2454x + 11.7182 | 9.43 |
| 1-Butanol | y = -4,222.7191x + 16.0093 | 6.29 |
| Aniline | y = -2,617.9524x + 11.8707 | 21.88 |
| 1-Methylnaphthalne | y = -3,478.5075x + 12.2994 | 1.87 |
| Acetic acid | y = -3,538.1534x + 13.5014 | 5.10 |
| 1-Chlorobutane | y = -6,061.7326x + 19.3203 | 0.36 |
| Triethylamine | y = -4,356.6959x + 15.8296 | 3.35 |
| Isobutyl acetate | y = -4,419.9958x + 16.6397 | 6.10 |
| 2-Butanone | y = -2,651.4361x + 11.8004 | 18.23 |
| 2-Methoxyethanol | y = -2,888.7237x + 12.9346 | 25.56 |
| Diethyl sulfide | y = -4,377.0711x + 15.9962 | 3.70 |
| 2-Butanol | y = -4,683.9333x + 17.5038 | 5.96 |
| 1,2-Dichloroethane | y = -5,565.2367x + 18.9389 | 1.30 |
| Methyl acetate | y = -3,414.6639x + 13.8299 | 10.71 |
| Anisole | y = -4,174.4368x + 15.0996 | 2.98 |
| Chloroform | y = -6,119.0096x + 22.1683 | 5.13 |
| Acetonitrile | y = -4,694.3149x + 16.1485 | 1.49 |
| 1-Bromobutane | y = -6,149.2304x + 19.8656 | 0.46 |
| 1,4-Dioxane | y = -2,748.1697x + 13.3681 | 63.18 |
| Toluene | y = -5,087.9485x + 16.9930 | 0.92 |
| Dimethylformamide | y = -4,844.2964x + 20.3419 | 59.49 |
| Methanol | y = -3,578.3400x + 13.6377 | 5.10 |
| 2-Propanol | y = -4,547.5633x + 17.0469 | 5.97 |
| Ethyl acetate | y = -3,873.9077x + 15.2625 | 9.61 |
| Acetone | y = -3,003.9520x + 12.8963 | 16.71 |
| Ethanol | y = -3,274.1819x + 13.0501 | 7.87 |

***Samples in ‘no crystal’ class***

Mefenamic acid did not crystallise at some supersaturation level in the following organic solvents listed in **Table S2**.

**Table S2** List of the solvents and their supersaturations in which mefenamic acid cannot crystallised within 5-day experimental period

| Solvent | Supersaturation ranges in which mefenamic acid cannot crystallise |
| --- | --- |
| 1-Bromobutane | 1.18 – 1.39 |
| 1-Butanol | 1.30 – 2.06 |
| 1-Chlorobutane | 1.12 – 1.28 |
| 1-Octanol | 1.26 – 1.41 |
| 2-Butanol | 1.12 – 1.39 |
| Aniline | 1.09 |
| Butyl acetate | 1.20 |
| Iodomethane | 1.24 |
| Isobutyl acetate | 1.10 – 1.30 |
| Toluene | 1.11 – 1.43 |
| Trichloroethylene | 1.09 – 1.45 |

***Dataset for random forest classification***

The dataset was established from 261 observations of crystal shapes obtained by cooling crystallisation from 30 different organic solvents. From this dataset, three models for the prediction of crystal shape were built. *Model 1* using the original dataset was used for the 3-class prediction. The outputs of the model were ‘polyhedral’, ‘needle’, and ‘no crystal’. The class of ‘no crystal’ was then removed from the dataset used in *Model 2* and *3* so that both models will be used for predicting only 2 crystal classes, which were ‘polyhedral’ and ‘needle’. The numbers of observations in the dataset used for *Model 1 – 3* are shown in **Table S3**.

**Table S3** Numbers of observations in the dataset used for training and testing each predictive model

| **Model** | **Number of observations** | | | |
| --- | --- | --- | --- | --- |
|  | **Polyhedral class** | **Needle class** | **No crystal class** | **Total** |
| Model 1 | 134 (51.3 %) | 83 (31.8 %) | 44 (16.9 %) | 261 |
| Model 2 | 134 (62.0 %) | 82 (38.0 %) | - | 216 |
| Model 3 | 82 (50.0 %) | 82 (50.0 %) | - | 164 |

***MOE molecular descriptors as the models’ variables***

Molecular descriptors can be defined as the molecular features in numerical form which represent the structural and chemical characteristics of a molecule. The 2D molecular descriptors are derived from solely the two-dimensional chemical structure without considering the conformation of the molecule. Some examples of 2D molecular descriptors range from simple attributes such as the number of atoms, the number of internal bonds, or molecular weight, to the properties such as atomic polarizabilities, molecular mass density, and LogP value. On the other hand, 3D molecular descriptors can be classified into 2 types, one depends on internal coordinates only and the other also depends on the absolute orientation of molecules.

Molecular descriptors can be calculated from various software. One of those is Molecular Operating Environment (MOE), the integrated computer-aided molecular design platform that operates various useful tools for visualizations, simulations, modelling, screening, including cheminformatics. Molecular descriptors calculated from MOE consist of two-dimensional and three-dimensional molecular descriptors. two-dimensional descriptors were divided into 7 categories, namely 1.) physical properties, 2.) subdivided surface areas, 3.) atom counts and bond counts, 4.) Kier & Hall connectivity and Kappa shape indices, 5.) adjacency and distance matrix, 6.) pharmacophore feature, 7.) partial charge. All 2D molecular descriptors applied in this work are shown in **Table S4**.

**Table S4** 2-D molecular descriptors

| **Descriptors** | **Category** | **Description** |
| --- | --- | --- |
| 2-D descriptors | | |
| apol, bpol, Fcharge, mr, SMR, Weight, logP (o/w), SlogP, vdw_vol, density, vdw-area | physical properties | Physical properties are calculated from the connection table of a molecule |
| SlogP_VSA0-SlogP_VSA9, SMR_VSA0 - SMR_VSA7 | subdivided surface areas | The Subdivided Surface Areas are descriptors based on an approximate accessible van der Waals surface area calculation for each atom, *vi* along with some other atomic property, *pi*. |
| a_aro, a_count, a_heavy, a_ICM, a_IC, a_nH, a_nB, a_nC, a_nN, a_nO, a_nF, a_nP, a_nS, a_nCl, a_nBr, a_nI, b_1rotN, b_1rotR, b_ar, b_count, b_double, b_heavy, b-rotN, b_rotR, b_single, b_triple, VAdjMa, VAdjEq | atom count and bond count | The atom count and bond count descriptors are functions of the counts of atoms and bonds |
| chi0, chi0_C, chi1, chi1_C, chi0v, chi0v_C, chi1v, chi1v_C, Kier1 - Kier3, KierA1 - KierA3, KierFlex, zagreb | Kier&Hall Connectivity and Kappa Shape Indices | The Kier and Hall kappa molecular shape indices compare the molecular graph with minimal and maximal molecular graphs and are intended to capture different aspects of molecular shape. |
| balabanJ, diameter, petitjean, radius, VDistEq, VDistMa, weinerPath, weinerPol | Adjacency and Distance Matrix Descriptors | The adjacency matrix, M, of a chemical structure is defined by the elements [Mij] where Mij is 1 if atoms i and j are bonded and zero otherwise. The distance matrix, D, for a chemical structure is defined by the elements [Dij] where Dij is the length of the shortest path from atoms i to j; zero is used if atoms i and j are not part of the same connected component. |
| a_acc, a_acid, a_base, a_don, a_hyd, vsa_acc, vsa_acid, vsa_base, vsa_don, vsa_hyd, vsa_other, vsa_pol | Pharmacophore Feature Descriptors | The Pharmacophore Atom Type descriptors consider only the heavy atoms of a molecule and assign a type to each atom |
| Q_PC+ PEOE_PC+, Q_PC- PEOE_PC-, Q_RPC+ PEOE_RPC+, Q_RPC- PEOE_RPC-, Q_VSA_POS  PEOE_VSA_POS,  PEOE_VSA_NEG,  PEOE_VSA_PPOS, PEOE_VSA_PNEG, PEOE_VSA_HYD,  PEOE_VSA_POL,  PEOE_VSA_FPOS, PEOE_VSA_FNEG, Q_VSA_FPPOS PEOE_VSA_FPPOS, Q_VSA_FPNEG PEOE_VSA_FPNEG, Q_VSA_FHYD PEOE_VSA_FHYD, Q_VSA_FPOL PEOE_VSA_FPOL, PEOE_VSA+6 - PEOE_VSA+0, PEOE_VSA-0 - PEOE_VSA-6 | Partial Charge Descriptors | Descriptors that depend on the partial charge of each atom of a chemical structure require calculation of those partial charges. |

To investigate if the different sets of solvent molecular descriptors affect the model performance, feature selection was then applied. Atom counts, bond counts, and pharmacophore feature descriptors were selected as the model’s variables in Model 32 – Model 59. Physical property descriptors were selected for Model 60 – Model 87. The details of the selected descriptors were listed in **Table S5**.

**Table S5** The list of physical properties, atom counts and bond counts, and pharmacophore feature solvent descriptors with codes and descriptions

| **Category** | **Descriptors** | **Descriptions** |
| --- | --- | --- |
| **Molecular structure and connectivity (second set of descriptors)** | a_aro | Number of aromatic atoms |
|  | a_count | Number of atoms |
|  | a_heavy | Number of heavy atoms |
|  | a_nH, a_nC, a_nN, a_nO, a_nS, a_nCl, a_nBr, a_nI | Number of hydrogen, carbon, nitrogen, oxygen, sulfur, chlorine, bromine, iodine atoms |
|  | b_ar | Number of aromatic bonds |
|  | b_count | Number of bonds |
|  | b_heavy | Number of bonds between heavy atoms |
|  | b_rotN | Number of rotatable bonds |
|  | b_single, b_double, b_triple | Number of single, double, and triple bonds |
|  | chiral | Number of chiral centres |
|  | opr_brigid | Number of rigid bonds |
|  | rings | Number of rings. |
|  | a_acc | Number of hydrogen bond acceptor atoms |
|  | a_acid | Number of acidic atoms |
|  | a_base | Number of basic atoms |
|  | a_don | Number of hydrogen bond donor atoms |
|  | a_hyd | Number of hydrophobic atoms |
| **Physical properties (third set of descriptors)** | apol | Sum of the atomic polarizabilities |
|  | bpol | Sum of the absolute value of the difference between atomic polarizabilities of all bonded atoms in the molecule |
|  | density | Molecular mass density |
|  | mr, SMR | Molecular refractivity |
|  | weight | Molecular weight |
|  | logP(o/w), SlogP | Log of the octanol/water partition coefficient |
|  | logS | Log of the aqueous solubility (mol/L) |
|  | reactive | Indicator of the presence of reactive groups |
|  | TPSA | Polar surface area |
|  | vdw_vol | van der Waals volume |
|  | vdw_area | Area of van der Waals surface |

***Crystal face indexing***

From the samples exhibited crystal shapes as a plate, the single crystal was mounted and analysed by single-crystal X-ray diffraction (SC-XRD). Based on face indexing data, the biggest face which dominated the polyhedral crystal is [100] (**Figure S1**). This observed crystal shape corresponded to the BFDH morphology of mefenamic acid crystal form-I (**Figure S2**).


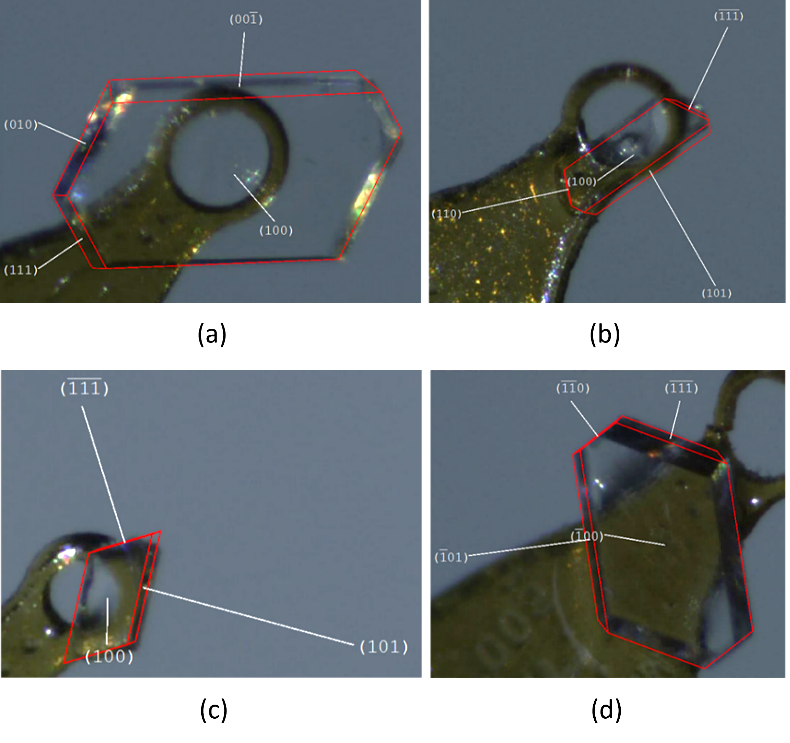


**Figure S1** Face indexing of single crystal of mefenamic acid crystallised from (a) methanol, (b) ethyl acetate, (c) acetonitrile, and (d) 2-butanol. The face that dominates crystal morphology is (100).


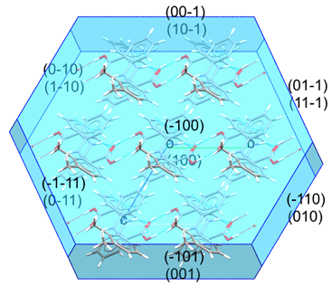


**Figure S2** BFDH morphology of mefenamic acid crystal form-I shows plate-like crystal morphology generated with Mercury software (version 2021.2.0)

***The performance of Model 1, Model 2, and Model 3***

Confusion matrixes of *Model 1*, *2*, and *3* are presented in **Figure 5a**, **Figure 5b**, and **Figure 5c**, respectively. The number in each column represents the number of the class predicted by the model, while the number in each row represents the number of experimental results or actual class in the dataset. Sum of the numbers in each column is the total number of the data in each predicted class, while sum of the numbers in each row is the total number of actual data in each class of the test set. The numbers on the diagonal axis of the matrix represent correct predictions. On the other hand, the numbers in the remaining fields represent incorrect predictions.


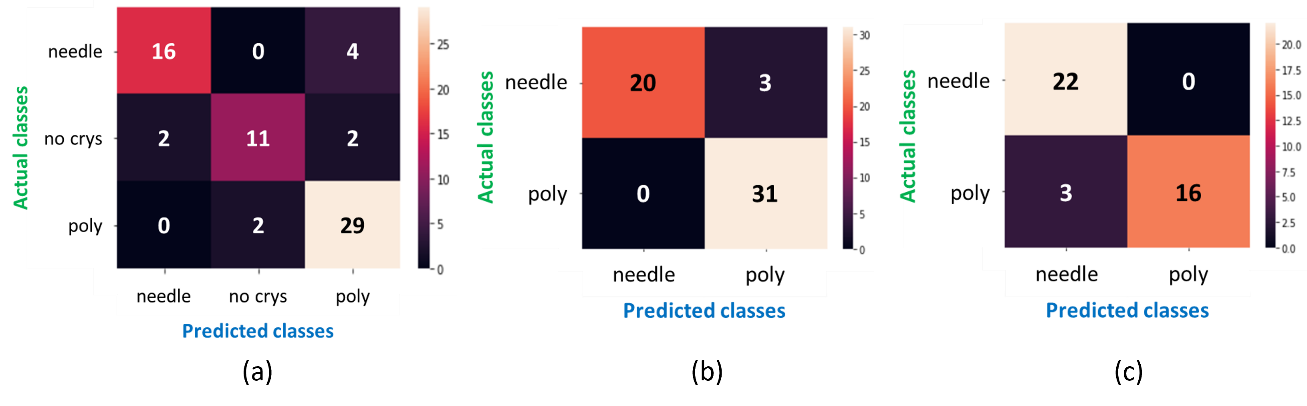


**Figure S3** The confusion matrix of random forest classification model for the prediction of mefenamic acid crystal shapes (a) 3-class prediction, (b) 2-class prediction with class-imbalance, and (c) 2-class prediction without class-imbalance

***Powder X-ray Diffraction***

All samples were checked by Powder X-ray Diffraction (PXRD) for checking polymorphic form. The powder patterns showed consistency with mefenamic acid form I (**Figure S4**).


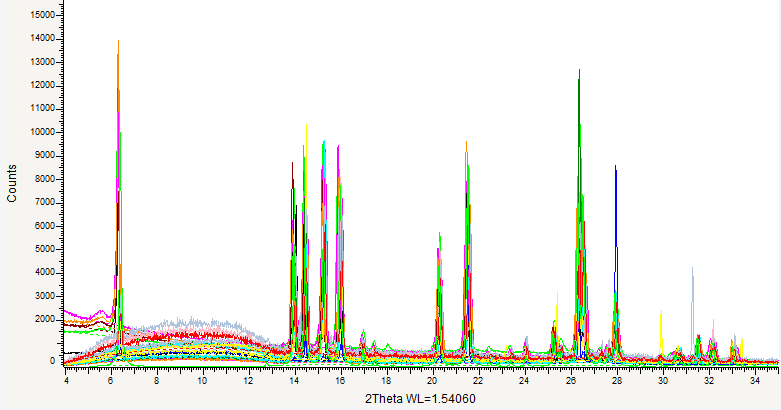


**Figure S4** Powder patterns of mefenamic acid crystals from the solvents studied in this work (except triethylamine and DMF). All patterns corresponded to mefenamic acid form-I

***Feature importance score ranking of random forest classification models***

The following table is an extension to Table 5 in the main paper. The importance score was provided for each molecular descriptor in all variable groups used for training Model 2. The variables in Group 1 and Group 2 presented in Table S6 are in the Top-15 ranking.

**Table S6** Important scores of the solvent molecular descriptors in the variables Group 1, 2, and 3 of the models for predicting the shape of mefenamic acid crystallised from individual solvents

| **Crystallisation solvent** | **Variable Group 1: All solvent  descriptors** | | **Variable Group 2: Atom count/bond count + pharmacophore features** | | **Variable Group 3: Physical properties** | |
| --- | --- | --- | --- | --- | --- | --- |
|  | **Descriptor** | **Important** | **Descriptor** | **Important** | **Descriptor** | **Important** |
|  |  | **score** |  | **score** |  | **score** |
| **Solvents where the crystals were 100% accurately predicted by the models** | | | | | | |
| **1,2-Dichloroethane** | rings | 0.0279 | opr_brigid | 0.0768 | logS | 0.1178 |
|  | BCUT_PEOE_2 | 0.0251 | a_count | 0.0705 | mr | 0.0883 |
|  | logS | 0.0205 | b_single | 0.0677 | bpol | 0.0868 |
|  | GCUT_PEOE_2 | 0.0199 | b_count | 0.0582 | vdw_vol | 0.0793 |
|  | BCUT_SMR_1 | 0.0198 | rings | 0.0569 | vdw_area | 0.0760 |
|  | GCUT_PEOE_1 | 0.0178 | a_nH | 0.0460 | logP(o/w) | 0.0647 |
|  | opr_brigid | 0.0153 | a_aro | 0.0417 | SMR | 0.0599 |
|  | logP(o/w) | 0.0148 | b_heavy | 0.0412 | apol | 0.0581 |
|  | PEOE_VSA-5 | 0.0127 | b_ar | 0.0406 | density | 0.0421 |
|  | BCUT_SLOGP_1 | 0.0126 | a_nBr | 0.0398 | SlogP | 0.0414 |
|  | BCUT_PEOE_1 | 0.0125 | a_hyd | 0.0392 | Weight | 0.0402 |
|  | h_log_pbo | 0.0124 | a_nO | 0.0373 | TPSA | 0.0346 |
|  | h_emd_C | 0.0123 | a_nC | 0.0322 | reactive | 0.0040 |
|  | a_aro | 0.0117 | a_heavy | 0.0306 |  |  |
|  | GCUT_SMR_0 | 0.0113 | a_acc | 0.0209 |  |  |
| **Chloroform** | BCUT_PEOE_2 | 0.0258 | opr_brigid | 0.0756 | logS | 0.1030 |
|  | rings | 0.0220 | b_single | 0.0747 | mr | 0.0835 |
|  | chi1_C | 0.0190 | rings | 0.0647 | vdw_vol | 0.0791 |
|  | GCUT_PEOE_2 | 0.0176 | a_count | 0.0616 | bpol | 0.0774 |
|  | opr_brigid | 0.0174 | b_count | 0.0557 | logP(o/w) | 0.0760 |
|  | b_max1len | 0.0162 | a_nC | 0.0479 | vdw_area | 0.0743 |
|  | GCUT_PEOE_1 | 0.0158 | a_nH | 0.0430 | SMR | 0.0740 |
|  | BCUT_SMR_1 | 0.0150 | a_nO | 0.0414 | apol | 0.0636 |
|  | logS | 0.0149 | a_nBr | 0.0387 | Weight | 0.0511 |
|  | logP(o/w) | 0.0146 | b_ar | 0.0343 | SlogP | 0.0466 |
|  | h_log_pbo | 0.0141 | a_aro | 0.0342 | density | 0.0395 |
|  | GCUT_PEOE_3 | 0.0138 | a_hyd | 0.0336 | TPSA | 0.0267 |
|  | BCUT_SLOGP_1 | 0.0138 | b_heavy | 0.0333 | reactive | 0.0040 |
|  | PEOE_VSA-5 | 0.0115 | a_heavy | 0.0329 |  |  |
|  | mr | 0.0113 | b_rotN | 0.0328 |  |  |
| **Ethanol** | BCUT_PEOE_2 | 0.0260 | opr_brigid | 0.0838 | logS | 0.0988 |
|  | rings | 0.0239 | b_single | 0.0709 | mr | 0.0873 |
|  | opr_brigid | 0.0222 | a_nH | 0.0626 | vdw_vol | 0.0849 |
|  | logS | 0.0197 | rings | 0.0602 | bpol | 0.0819 |
|  | GCUT_PEOE_1 | 0.0177 | a_count | 0.0597 | vdw_area | 0.0730 |
|  | GCUT_PEOE_2 | 0.0145 | b_count | 0.0589 | logP(o/w) | 0.0700 |
|  | chi1_C | 0.0141 | a_nO | 0.0405 | SlogP | 0.0632 |
|  | GCUT_SLOGP_3 | 0.0138 | a_hyd | 0.0377 | SMR | 0.0620 |
|  | PEOE_VSA-5 | 0.0138 | a_nBr | 0.0346 | apol | 0.0542 |
|  | BCUT_PEOE_1 | 0.0128 | b_heavy | 0.0340 | Weight | 0.0445 |
|  | BCUT_SMR_1 | 0.0126 | a_nC | 0.0333 | density | 0.0418 |
|  | h_log_pbo | 0.0122 | b_ar | 0.0330 | TPSA | 0.0295 |
|  | GCUT_SMR_0 | 0.0120 | a_aro | 0.0326 | reactive | 0.0045 |
|  | mr | 0.0118 | a_heavy | 0.0289 |  |  |
|  | BCUT_SLOGP_1 | 0.0117 | b_rotN | 0.0278 |  |  |
| **Trichloroethylene** | BCUT_PEOE_2 | 0.0290 | opr_brigid | 0.0824 | logS | 0.1148 |
|  | rings | 0.0254 | b_single | 0.0713 | mr | 0.0938 |
|  | GCUT_PEOE_1 | 0.0229 | rings | 0.0680 | bpol | 0.0921 |
|  | opr_brigid | 0.0205 | a_count | 0.0649 | logP(o/w) | 0.0761 |
|  | logS | 0.0190 | b_count | 0.0533 | SMR | 0.0699 |
|  | GCUT_PEOE_2 | 0.0179 | a_nH | 0.0439 | vdw_vol | 0.0698 |
|  | BCUT_PEOE_1 | 0.0173 | a_nO | 0.0418 | vdw_area | 0.0620 |
|  | BCUT_SLOGP_1 | 0.0154 | a_nBr | 0.0409 | apol | 0.0576 |
|  | BCUT_SLOGP_2 | 0.0152 | a_hyd | 0.0397 | SlogP | 0.0464 |
|  | BCUT_SMR_1 | 0.0145 | b_heavy | 0.0365 | Weight | 0.0441 |
|  | b_max1len | 0.0136 | a_aro | 0.0354 | density | 0.0348 |
|  | mr | 0.0136 | a_heavy | 0.0331 | TPSA | 0.0336 |
|  | h_log_pbo | 0.0132 | b_ar | 0.0325 | reactive | 0.0046 |
|  | PEOE_VSA-5 | 0.0131 | b_rotN | 0.0313 |  |  |
|  | chi1_C | 0.0130 | a_nC | 0.0281 |  |  |
| **Aniline** | BCUT_PEOE_2 | 0.0245 | b_single | 0.0719 | logS | 0.1014 |
|  | rings | 0.0242 | opr_brigid | 0.0681 | bpol | 0.0947 |
|  | opr_brigid | 0.0227 | b_count | 0.0590 | SMR | 0.0718 |
|  | logS | 0.0181 | rings | 0.0584 | mr | 0.0709 |
|  | GCUT_PEOE_1 | 0.0156 | a_count | 0.0578 | logP(o/w) | 0.0688 |
|  | b_max1len | 0.0155 | a_hyd | 0.0462 | vdw_area | 0.0676 |
|  | GCUT_SLOGP_3 | 0.0154 | a_nBr | 0.0398 | SlogP | 0.0651 |
|  | BCUT_SMR_3 | 0.0153 | a_nH | 0.0396 | apol | 0.0616 |
|  | BCUT_SMR_1 | 0.0136 | a_nO | 0.0379 | vdw_vol | 0.0612 |
|  | PEOE_VSA-5 | 0.0129 | b_heavy | 0.0360 | density | 0.0463 |
|  | h_log_pbo | 0.0127 | b_rotN | 0.0359 | Weight | 0.0445 |
|  | h_emd_C | 0.0111 | a_nC | 0.0356 | TPSA | 0.0346 |
|  | GCUT_PEOE_2 | 0.0108 | a_aro | 0.0300 | reactive | 0.0020 |
|  | PEOE_VSA+5 | 0.0106 | a_heavy | 0.0293 |  |  |
|  | GCUT_PEOE_3 | 0.0099 | b_ar | 0.0259 |  |  |
| **Anisole** | rings | 0.0221 | opr_brigid | 0.0684 | logS | 0.0940 |
|  | GCUT_PEOE_1 | 0.0188 | b_single | 0.0661 | mr | 0.0905 |
|  | BCUT_PEOE_2 | 0.0187 | a_count | 0.0637 | logP(o/w) | 0.0804 |
|  | GCUT_SLOGP_3 | 0.0163 | b_count | 0.0578 | bpol | 0.0744 |
|  | opr_brigid | 0.0163 | rings | 0.0491 | SMR | 0.0671 |
|  | BCUT_PEOE_1 | 0.0163 | a_nH | 0.0468 | vdw_area | 0.0647 |
|  | PEOE_PC- | 0.0162 | a_nBr | 0.0456 | vdw_vol | 0.0633 |
|  | GCUT_PEOE_2 | 0.0159 | a_nO | 0.0449 | Weight | 0.0559 |
|  | logS | 0.0156 | a_nC | 0.0426 | density | 0.0489 |
|  | chi1_C | 0.0150 | b_rotN | 0.0388 | TPSA | 0.0487 |
|  | PEOE_VSA-5 | 0.0145 | b_heavy | 0.0380 | SlogP | 0.0470 |
|  | logP(o/w) | 0.0137 | a_hyd | 0.0360 | apol | 0.0462 |
|  | PEOE_PC+ | 0.0127 | a_aro | 0.0318 | reactive | 0.0052 |
|  | h_ema | 0.0124 | b_ar | 0.0270 |  |  |
|  | a_IC | 0.0120 | a_acc | 0.0249 |  |  |
| **Toluene** | BCUT_PEOE_2 | 0.0235 | b_single | 0.0748 | logS | 0.1033 |
|  | b_max1len | 0.0217 | opr_brigid | 0.0696 | mr | 0.0841 |
|  | rings | 0.0208 | rings | 0.0668 | bpol | 0.0774 |
|  | logS | 0.0198 | a_count | 0.0630 | vdw_vol | 0.0737 |
|  | GCUT_PEOE_1 | 0.0170 | b_count | 0.0524 | vdw_area | 0.0722 |
|  | PEOE_VSA-5 | 0.0168 | a_nO | 0.0448 | logP(o/w) | 0.0689 |
|  | opr_brigid | 0.0166 | a_nBr | 0.0403 | SMR | 0.0642 |
|  | chi1_C | 0.0157 | a_nH | 0.0401 | apol | 0.0581 |
|  | h_ema | 0.0151 | a_hyd | 0.0384 | SlogP | 0.0531 |
|  | GCUT_PEOE_2 | 0.0151 | b_rotN | 0.0380 | density | 0.0482 |
|  | BCUT_PEOE_1 | 0.0140 | b_heavy | 0.0349 | Weight | 0.0470 |
|  | GCUT_PEOE_3 | 0.0125 | a_heavy | 0.0342 | TPSA | 0.0401 |
|  | BCUT_SLOGP_1 | 0.0124 | a_aro | 0.0328 | reactive | 0.0036 |
|  | h_log_pbo | 0.0116 | a_nC | 0.0309 |  |  |
|  | h_emd_C | 0.0106 | b_ar | 0.0274 |  |  |
| **Solvents where the crystals were inaccurately predicted by the models** | | | | | | |
| **1-Chlorobutane** | rings | 0.0276 | opr_brigid | 0.0815 | logS | 0.1159 |
|  | BCUT_SMR_1 | 0.0201 | b_single | 0.0788 | bpol | 0.0838 |
|  | GCUT_PEOE_1 | 0.0194 | a_count | 0.0654 | vdw_vol | 0.0778 |
|  | BCUT_PEOE_2 | 0.0191 | a_nH | 0.0590 | mr | 0.0774 |
|  | opr_brigid | 0.0186 | b_count | 0.0580 | logP(o/w) | 0.0746 |
|  | logS | 0.0177 | a_hyd | 0.0547 | vdw_area | 0.0724 |
|  | GCUT_PEOE_2 | 0.0169 | rings | 0.0537 | SMR | 0.0662 |
|  | SMR_VSA5 | 0.0163 | a_nO | 0.0454 | apol | 0.0597 |
|  | PEOE_VSA-5 | 0.0157 | a_nC | 0.0409 | density | 0.0446 |
|  | GCUT_PEOE_3 | 0.0147 | a_aro | 0.0331 | Weight | 0.0442 |
|  | BCUT_SLOGP_1 | 0.0136 | b_ar | 0.0315 | SlogP | 0.0384 |
|  | a_aro | 0.0126 | b_heavy | 0.0314 | TPSA | 0.0321 |
|  | GCUT_SLOGP_3 | 0.0120 | b_rotN | 0.0270 | reactive | 0.0034 |
|  | h_log_pbo | 0.0119 | a_heavy | 0.0265 |  |  |
|  | BCUT_PEOE_1 | 0.0113 | a_nBr | 0.0254 |  |  |
| **1-Octanol** | chi1_C | 0.0287 | b_heavy | 0.0806 | logS | 0.1161 |
|  | zagreb | 0.0280 | opr_brigid | 0.0727 | vdw_vol | 0.1032 |
|  | rings | 0.0211 | b_single | 0.0602 | mr | 0.0973 |
|  | GCUT_SMR_3 | 0.0207 | a_count | 0.0536 | logP(o/w) | 0.0749 |
|  | GCUT_SLOGP_3 | 0.0192 | b_count | 0.0496 | SMR | 0.0607 |
|  | logP(o/w) | 0.0184 | a_hyd | 0.0486 | apol | 0.0606 |
|  | radius | 0.0177 | rings | 0.0466 | bpol | 0.0580 |
|  | opr_brigid | 0.0174 | a_nBr | 0.0431 | SlogP | 0.0579 |
|  | GCUT_PEOE_1 | 0.0172 | a_nC | 0.0400 | Weight | 0.0455 |
|  | b_heavy | 0.0165 | a_nH | 0.0366 | vdw_area | 0.0447 |
|  | logS | 0.0161 | a_nO | 0.0365 | density | 0.0408 |
|  | BCUT_PEOE_2 | 0.0148 | a_aro | 0.0326 | TPSA | 0.0337 |
|  | mr | 0.0145 | b_rotN | 0.0322 | reactive | 0.0036 |
|  | BCUT_SMR_3 | 0.0143 | a_heavy | 0.0319 |  |  |
|  | VAdjMa | 0.0131 | b_ar | 0.0252 |  |  |
| **Triethylamine** | GCUT_SMR_3 | 0.0360 | b_single | 0.0819 | mr | 0.1025 |
|  | GCUT_SLOGP_3 | 0.0258 | opr_brigid | 0.0720 | vdw_vol | 0.0987 |
|  | mr | 0.0204 | rings | 0.0657 | logS | 0.0985 |
|  | SMR | 0.0196 | a_count | 0.0606 | SMR | 0.0768 |
|  | apol | 0.0189 | b_count | 0.0571 | apol | 0.0762 |
|  | BCUT_PEOE_2 | 0.0179 | a_nC | 0.0456 | bpol | 0.0588 |
|  | b_max1len | 0.0177 | a_heavy | 0.0441 | logP(o/w) | 0.0584 |
|  | GCUT_PEOE_1 | 0.0173 | a_nH | 0.0438 | SlogP | 0.0569 |
|  | logS | 0.0156 | a_hyd | 0.0431 | vdw_area | 0.0511 |
|  | opr_brigid | 0.0153 | b_heavy | 0.0424 | Weight | 0.0410 |
|  | GCUT_PEOE_3 | 0.0151 | a_nO | 0.0357 | density | 0.0382 |
|  | vdw_vol | 0.0150 | a_nBr | 0.0337 | TPSA | 0.0352 |
|  | zagreb | 0.0140 | a_aro | 0.0294 | reactive | 0.0038 |
|  | PEOE_VSA-5 | 0.0139 | b_rotN | 0.0294 |  |  |
|  | BCUT_SMR_1 | 0.0136 | b_ar | 0.0240 |  |  |
| **Methyl acetate** | GCUT_SLOGP_3 | 0.0309 | opr_brigid | 0.1000 | vdw_vol | 0.1065 |
|  | BCUT_PEOE_2 | 0.0304 | rings | 0.0863 | mr | 0.1050 |
|  | rings | 0.0229 | a_count | 0.0570 | apol | 0.0938 |
|  | chi1_C | 0.0211 | b_single | 0.0545 | vdw_area | 0.0886 |
|  | mr | 0.0194 | a_nC | 0.0533 | SMR | 0.0795 |
|  | GCUT_PEOE_2 | 0.0173 | a_hyd | 0.0473 | bpol | 0.0679 |
|  | opr_brigid | 0.0168 | b_ar | 0.0451 | logS | 0.0636 |
|  | GCUT_PEOE_1 | 0.0167 | b_count | 0.0411 | logP(o/w) | 0.0517 |
|  | BCUT_SMR_3 | 0.0166 | a_aro | 0.0396 | Weight | 0.0381 |
|  | radius | 0.0163 | a_nBr | 0.0371 | TPSA | 0.0318 |
|  | GCUT_SMR_3 | 0.0161 | a_nH | 0.0339 | density | 0.0317 |
|  | BCUT_SLOGP_1 | 0.0150 | b_heavy | 0.0325 | SlogP | 0.0296 |
|  | a_aro | 0.0140 | a_nO | 0.0277 | reactive | 0.0031 |
|  | GCUT_PEOE_3 | 0.0139 | a_heavy | 0.0248 |  |  |
|  | vdw_vol | 0.0135 | b_rotN | 0.0231 |  |  |
| **Nitromethane** | BCUT_PEOE_2 | 0.0277 | opr_brigid | 0.0860 | bpol | 0.1013 |
|  | rings | 0.0259 | a_count | 0.0763 | logS | 0.0969 |
|  | GCUT_PEOE_3 | 0.0198 | b_count | 0.0721 | vdw_vol | 0.0954 |
|  | logS | 0.0177 | b_single | 0.0687 | SMR | 0.0842 |
|  | GCUT_PEOE_2 | 0.0165 | rings | 0.0681 | mr | 0.0764 |
|  | opr_brigid | 0.0156 | a_nBr | 0.0417 | apol | 0.0756 |
|  | BCUT_SMR_1 | 0.0153 | a_aro | 0.0403 | vdw_area | 0.0748 |
|  | BCUT_SLOGP_1 | 0.0147 | b_heavy | 0.0394 | logP(o/w) | 0.0549 |
|  | chi1_C | 0.0145 | a_nH | 0.0393 | density | 0.0459 |
|  | BCUT_PEOE_1 | 0.0140 | a_heavy | 0.0385 | SlogP | 0.0384 |
|  | GCUT_SMR_1 | 0.0140 | a_hyd | 0.0369 | Weight | 0.0345 |
|  | h_log_pbo | 0.0140 | b_ar | 0.0364 | TPSA | 0.0190 |
|  | mr | 0.0130 | a_nC | 0.0359 | reactive | 0.0024 |
|  | GCUT_SMR_3 | 0.0130 | b_rotN | 0.0327 |  |  |
|  | apol | 0.0124 | a_nO | 0.0192 |  |  |

**Table S7** The prediction accuracy of the models using supersaturation and an additional solvent descriptor as the model variables. The training dataset consists of the samples crystallised from all solvents as used in Model 2. This table shows how the addition of the additional variable contributed to model performance with the variable that improved the accuracy the most listed first (mr) to the variable that improved the accuracy the least (reactive) at the bottom.

| **Using SS and the variable listed below** | **4-fold CV Accuracy** |
| --- | --- |
| mr | 83.54 % |
| logS | 82.32 % |
| apol | 81.71 % |
| SMR | 81.10 % |
| logP(o/w) | 75.00 % |
| Weight | 74.39 % |
| bpol | 73.17 % |
| density | 73.17 % |
| vdw_vol | 70.73 % |
| vdw_area | 70.73 % |
| SlogP | 65.85 % |
| TPSA | 62.80 % |
| reactive | 59.15 % |
| no additional variable, SS only | 47.56 % |

***DSC Results***

According to the DSC results from the work carried out by Adam, et al. (2000)^30^, the onset temperature of the first endothermic peak of mefenamic acid ranges from 187°C to 205°C, and corresponds to the transition temperature from mefenamic acid form-I to form-II. A second sharper peak has an onset temperature of around 231 °C and is known to correspond to the melting point of mefenamic acid form-II.

For our mefenamic acid crystals crystallised from triethylamine, the DSC thermogram (Figure 5) showed different onset temperatures for both peaks when compared to the literature values. In our results, the first peak and the second peak have onset temperatures of 110.9 °C and 214.5 °C, respectively.


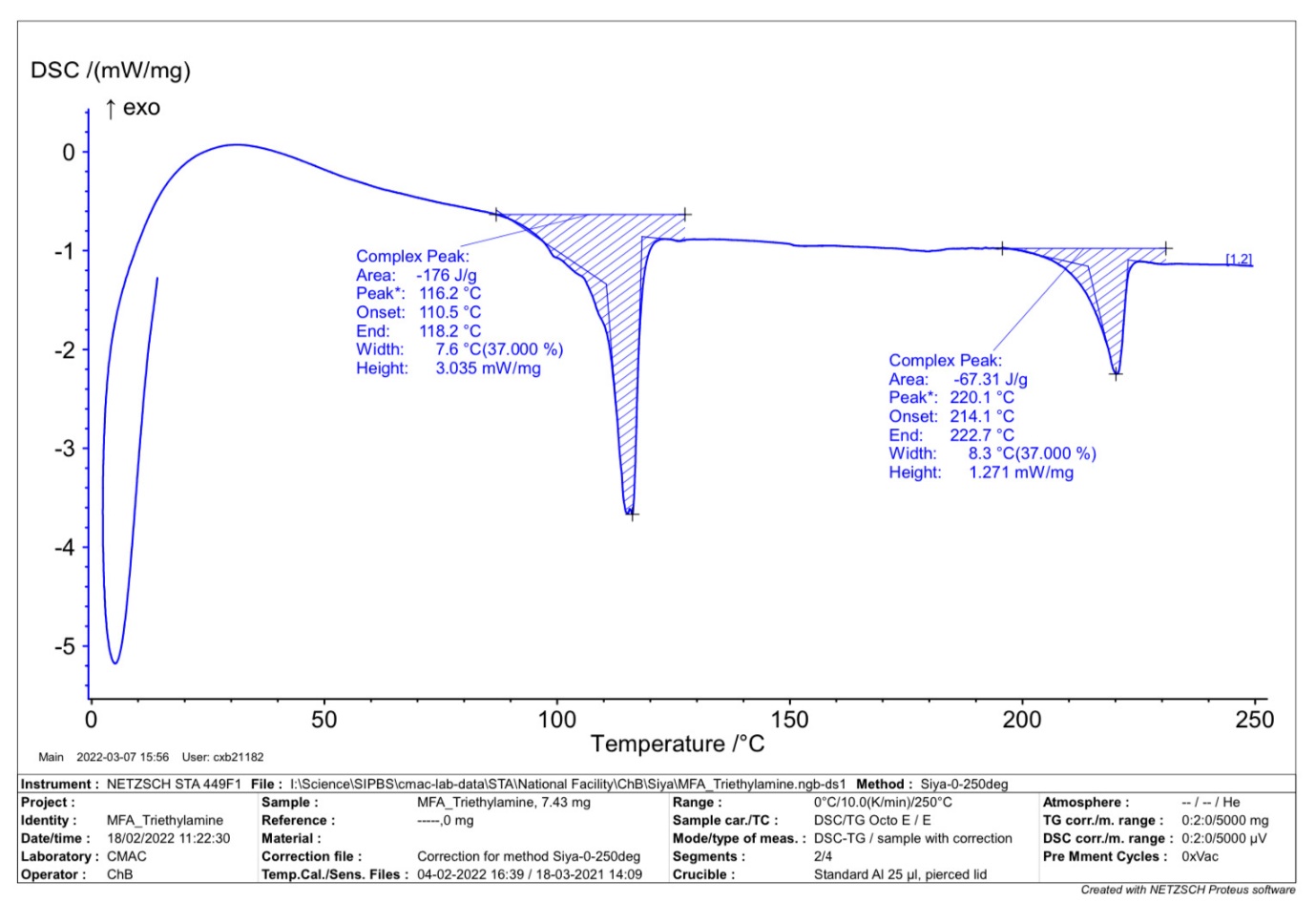


**Figure S5.** DSC curve for mefenamic acid crystallised from triethylamine by cooling crystallisation

While all other crystals grown in other solvents for which morphologies were poorly predicted by RF models were shown to be form I, the results shown here suggest that the poor prediction accuracy for the trimethylamine crystal morphology may be due to the distinctiveness of these crystals from the four previously document forms of mefenamic acid crystals rather than an innate flaw in the RF classification approach.
